# Supplementary material for: DMRforPairs: identifying Differentially Methylated Regions between unique samples using array based methylation profiles
Source: BMC Bioinformatics. 2014 May 15;15:141. doi: 10.1186/1471-2105-15-141 (PMC4046028; doi:10.1186/1471-2105-15-141)
Supplement: Additional file 2 — DMRforPairs output for the comparison of A431-MCF7 and NA17018-NA17105. Please start from the HTML files in each folder. Available via the BMC Bioinformatics website. [file 1471-2105-15-141-S2.zip › 1394847754114233_MOESM2_ESM/A431_MCF7/figures/11496.pdf]

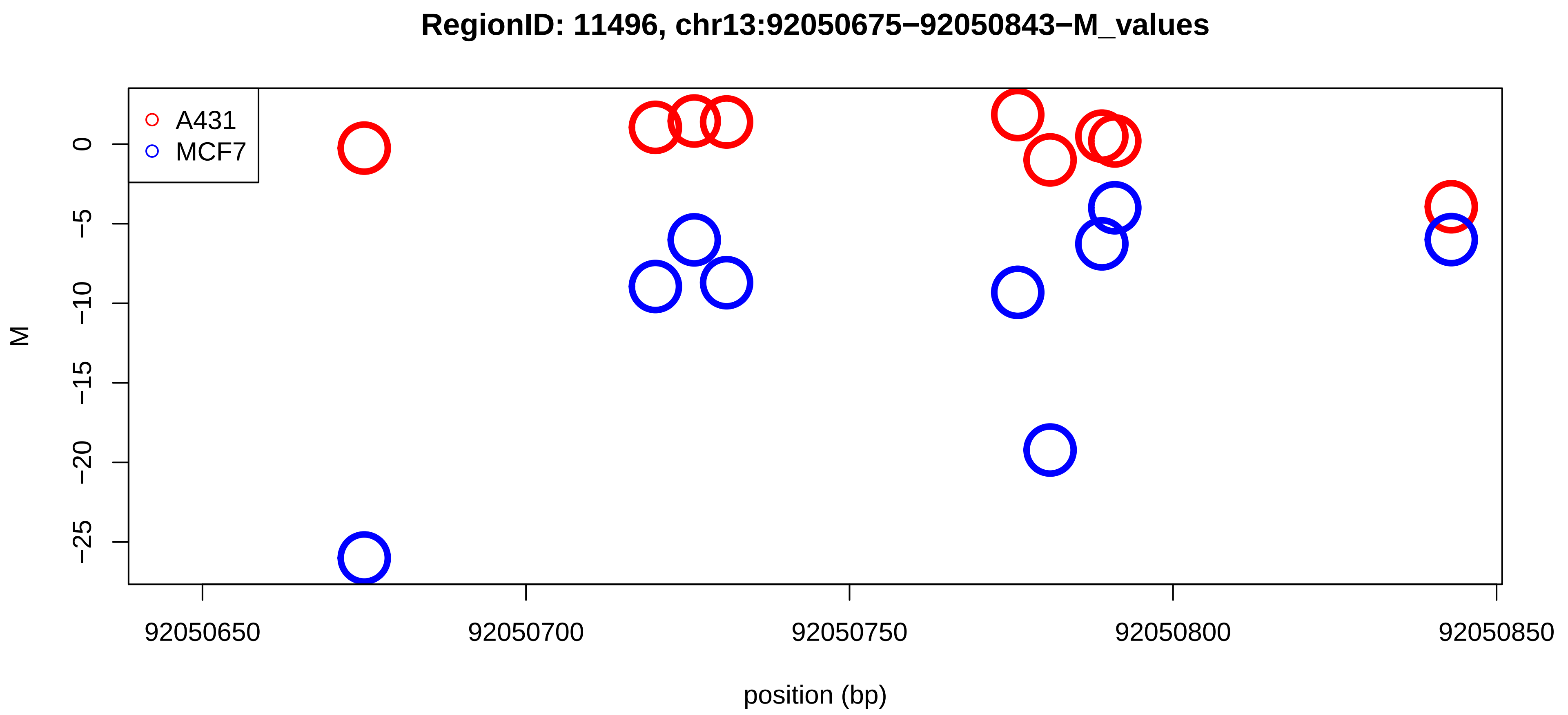

RegionID: 11496, chr13:92050675−92050843−M_values
A431
MCF7
M
0
−5
−10
−15
−20
−25
92050650
92050700
92050750
92050800
92050850
position (bp)

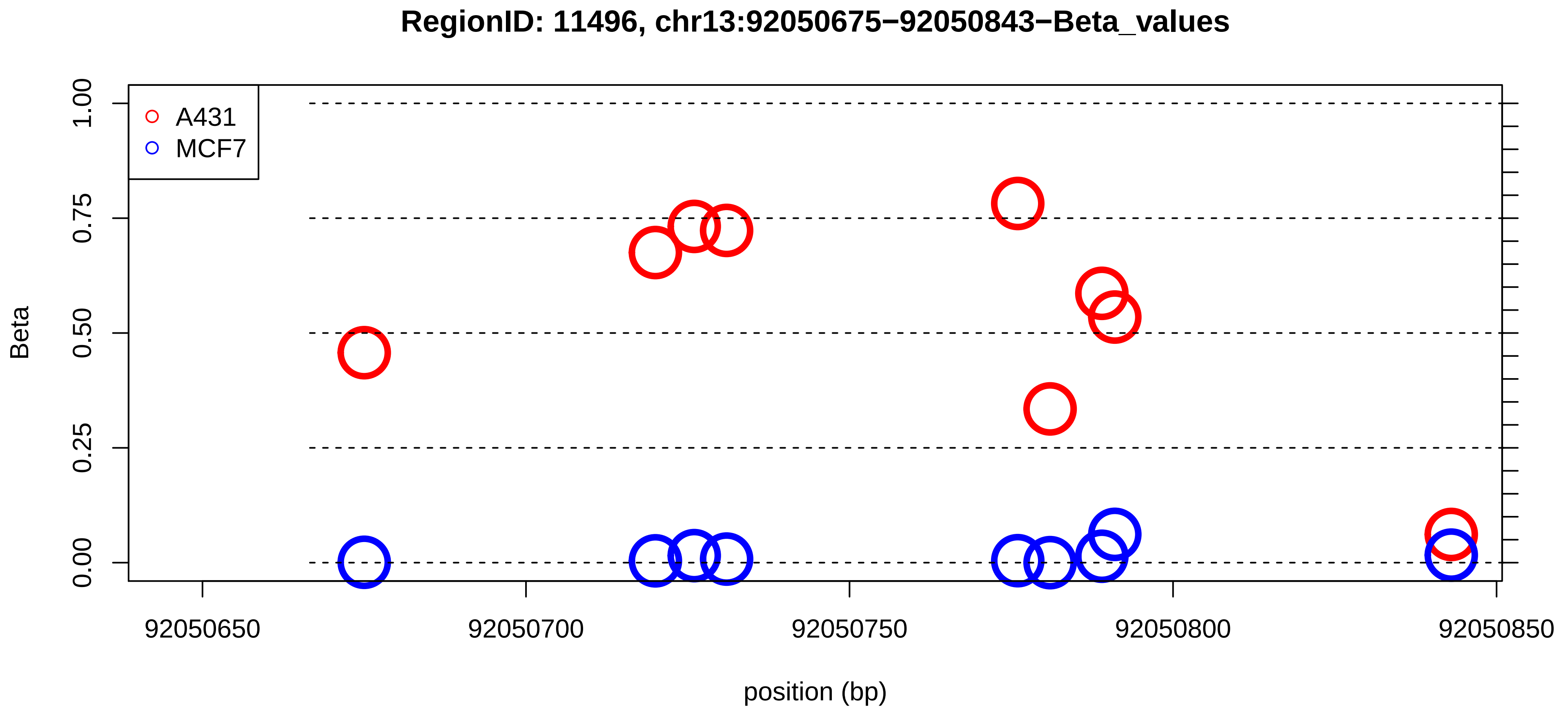

RegionID: 11496, chr13:92050675−92050843−Beta_values
A431
MCF7
Beta
0.00
0.25
0.50
0.75
1.00
92050650
92050700
92050750
92050800
92050850
position (bp)

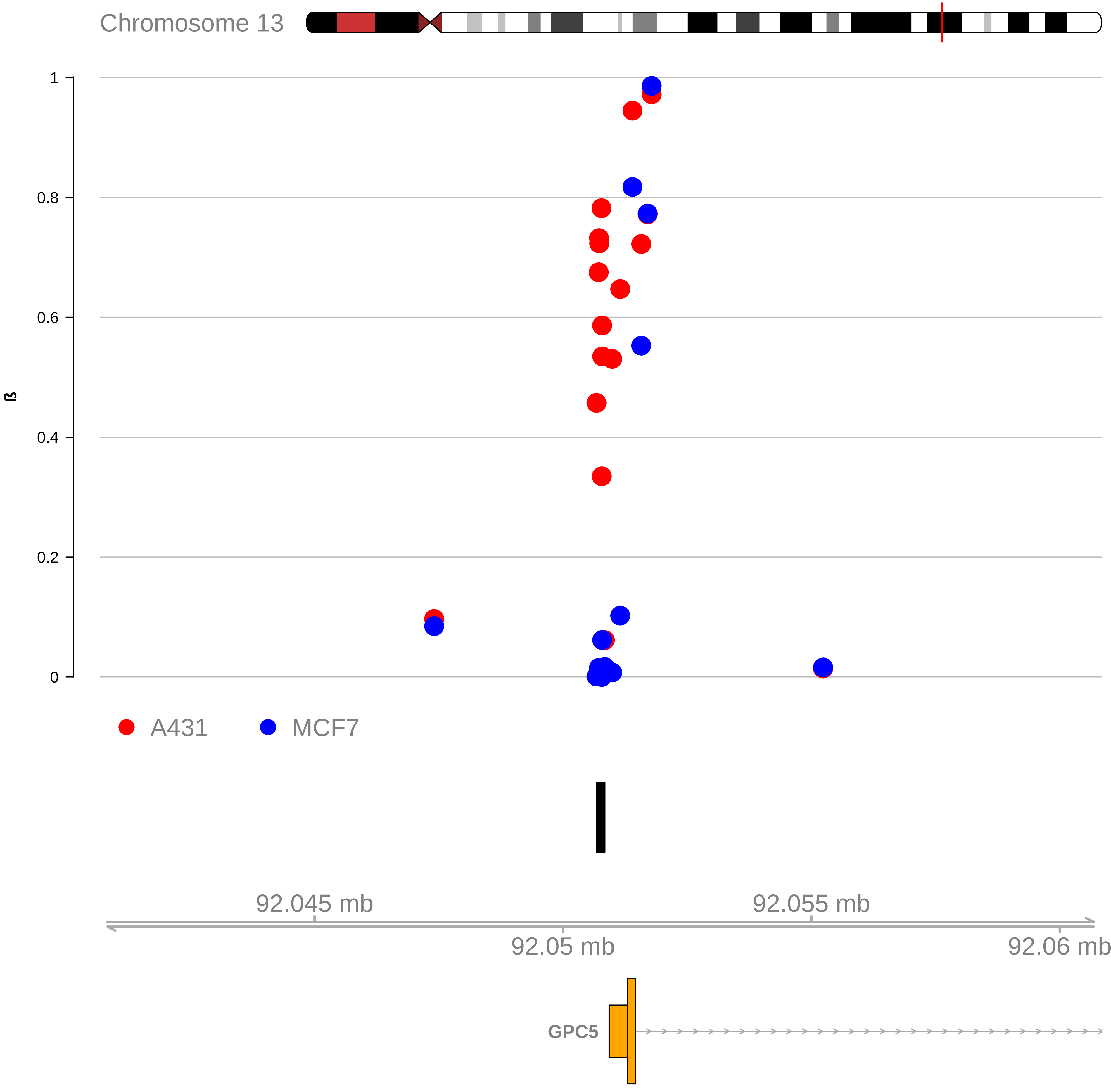
Chromosome 13
1
0.8
0.6
0.4
0.2
0
ß
A431
MCF7
92.045 mb
92.055 mb
92.05 mb
92.06 mb
GPC5
